# Cornelia de Lange syndrome and cancer: An open question

**DOI:** 10.1002/ajmg.a.62992

**Published:** 2022-10-17

**Authors:** Maria M. Pallotta, Maddalena Di Nardo, Raoul C. M. Hennekam, Frank J. Kaiser, Ilaria Parenti, Juan Pié, Feliciano J. Ramos, Antonie D. Kline, Antonio Musio

**Affiliations:** ^1^ Institute for Biomedical Technologies National Research Council Pisa Italy; ^2^ Department of Pediatrics, Emma Children's Hospital, Amsterdam UMC University of Amsterdam Amsterdam The Netherlands; ^3^ Institute for Human Genetics University Hospital Essen, University of Duisburg‐Essen Essen Germany; ^4^ Essen Center for Rare Diseases (Essener Zentrum für Seltene Erkrankungen, EZSE) University Hospital Essen Essen Germany; ^5^ Unit of Clinical Genetics and Functional Genomics, Department of Pharmacology‐Physiology, School of Medicine University of Zaragoza, CIBERER‐GCV02 and ISS‐Aragon Zaragoza Spain; ^6^ Clinical Genetics Unit, Service of Paediatrics University Hospital “Lozano Blesa”, University of Zaragoza, CIBERER GCV02 and ISS‐Aragón Zaragoza Spain; ^7^ Harvey Institute for Human Genetics Greater Baltimore Medical Center Baltimore Maryland USA

To the Editor,

Cohesin is an evolutionarily conserved protein complex implicated in all biological processes involving chromatin and chromosomes, such as replication, recombination, repair, transcription, and chromatin remodeling. Somatic variants in cohesin genes are associated with several types of cancer (Di Nardo et al., 2022), whereas germline variants are responsible for a class of human rare diseases currently called disorders of transcriptional regulation (DTRs) (Izumi, 2016), previously known as “cohesinopathies.” Cornelia de Lange syndrome (CdLS, OMIM #122470, #300590, #610759, #614701, #300882), with an estimated incidence of between 1:10,000 and 1:30,000 live births, is the most frequent among DTRs (Ramos et al., 2015). CdLS is a dominant condition characterized by multiple structural and physiological anomalies including microcephaly, facial dysmorphism, growth retardation, upper limb malformations, and neurodevelopmental delay (Kline et al., 2018). CdLS is caused by pathogenic variants in cohesin structural and regulatory genes, namely *NIPBL*, *SMC1A*, *SMC3*, *HDAC8*, *RAD21*, *BRD4*, and *ANKRD11* (Sarogni et al., 2020). CdLS cell lines show genome instability (Cukrov et al., 2018; Pallotta et al., 2021) and CdLS‐causative variants confer sensitivity to genotoxic treatments, suggesting that cohesin pathogenic variants impair DNA repair (Revenkova et al., 2009; Vrouwe et al., 2007). Variants in genes responsible for maintaining genome integrity are causative for human diseases such as Fanconi Anemia, Bloom syndrome, Werner syndrome, Ataxia Telangiectasia, and others, which are all characterized by cancer predisposition (Keijzers et al., 2017; Terabayashi & Hanada, 2018). Until now, no systematic study had been performed to investigate whether CdLS patients are predisposed to cancer. To gain new insight into the relationship between CdLS and cancer, we performed a systematic review of published literature listed in PubMed (https://pubmed.ncbi.nlm.nih.gov/). We manually checked 1267 manuscripts published from 1980 to 2022, as of August 2022. Moreover, the search was refined by using the strings “Cornelia de Lange syndrome and cancer,” “Cornelia de Lange syndrome and Barrett's esophagus,” Cornelia de Lange syndrome and Wilms tumor” as keywords search. In addition, we sent a questionnaire to the 10 main clinical groups and laboratories working on CdLS and five of them participated in this study. By these approaches, 17 manuscripts dealing with CdLS and cancer were found and 29 patients with clinical and/or molecular diagnosis of CdLS were identified with concomitant cancer development (Table 1). Most cancers (15 of 29, 51.7%) were related to the esophagus. Most of them were Barrett's esophagus (13 of 15, BE). BE is a premalignant condition that occurs when stratified squamous‐type mucosa of the lower esophagus is replaced by intestinal‐type columnar mucosa. It is thought that most esophageal adenocarcinomas (EA), a lethal malignancy with poor survival, arise from underlying BE tissue. This notion is supported by the observation that two cases of EA were described (DuVall & Walden, 1996; Macchini et al., 2010). Furthermore, three patients developed Wilms tumor, two developed leukemia, and two patients developed endometrial carcinoma, one of which had associated pancreatic neuroendocrine cancer (Table 1). A molecular diagnosis was available for a few of the patients with cancer development. For these patients, all identified causative variants affect *NIPBL*. Their phenotype ranges from mild to severe, suggesting that no correlation exists between CdLS phenotype and cancer development. NIPBL, a 316 kDa protein, is essential to load cohesin onto chromatin in collaboration with its molecular partner, MAU2. In addition, it is necessary to stimulate cohesin's ATPase activity, for chromatin looping, and it is crucial for cohesin's ability to extrude DNA into loops (Davidson & Peters, 2021; Davidson et al., 2019; Kim et al., 2019). The five detected *NIPBL* pathogenic variants are unique to each condition (Table 1, Figure 1). The variant associated with acute megakaryoblastic leukemia maps in the acceptor splice site of intron 36, whereas a deletion of *NIPBL* gene was identified in a sacrococcygeal teratoma. The last three *NIPBL* pathogenic variants are two missense substitutions and an out‐of‐frame duplication, which were identified in Wilms tumor and pancreatic neuroendocrine cancer and in acute lymphoblastic leukemia, respectively. Most variants cause a predicted truncated protein that likely leads to a partial reduction in NIPBL production, resulting in haploinsufficiency.

**TABLE 1:** CdLS patients with concomitant cancer development

Cancer type	Number of patients	CdLS‐causative gene	Variant	Effect	Age of cancer diagnosis	References
Acute lymphoblastic leukemia	1	*NIPBL*	c.7977dupT	Premature truncation	8 years	Fazio et al. (2019)
Acute megakaryoblastic leukemia	1	*NIPBL*	c.6344–2A>G	Premature truncation	3 years	Vial et al. (2018)
Esophagus (premalignant Barrett's esophagus, adenocarcinoma)	15	Unknown				DuVall & Walden (1996); Kline et al. (2007); Luzzani et al. (2003); Macchini et al. (2010); Pei et al. (2000); Schrier et al. (2011)
Choroid plexus papilloma	1	Unknown				Chico‐Ponce de León et al. (2015)
Endometrial carcinoma	1	Unknown				Tate et al. (2019)
Gastric cancer	1	Unknown				Schrier et al. (2011)
Intracranial germinoma	1	Unknown				Sato et al. (1986)
Lymphoma	1	Unknown				Schrier et al. (2011)
Liver haemangioendothelioma	1	Unknown				Maruiwa et al. (1988)
Pancreatic neuroendocrine/endometrial cancer	1	*NIPBL*	c.620 C>G	Premature truncation	27 years	Wright et al. (2022)
Sacrococcygeal teratoma	1	*NIPBL*	Exons 42–47 deletion	Amino acids deletion	20 weeks of gestation	Banait et al. (2015)
Suprasellar germinoma	1	Unknown				Sugita et al. (1986)
Wilms tumor	3	*NIPBL* Unknown	c.4920 G>A	Splice alteration	4 years	Charles et al. (1997); Maruiwa et al. (1988); Santoro et al. (2016)

Abbreviation: CdLS, Cornelia de Lange syndrome.

**FIGURE 1:**
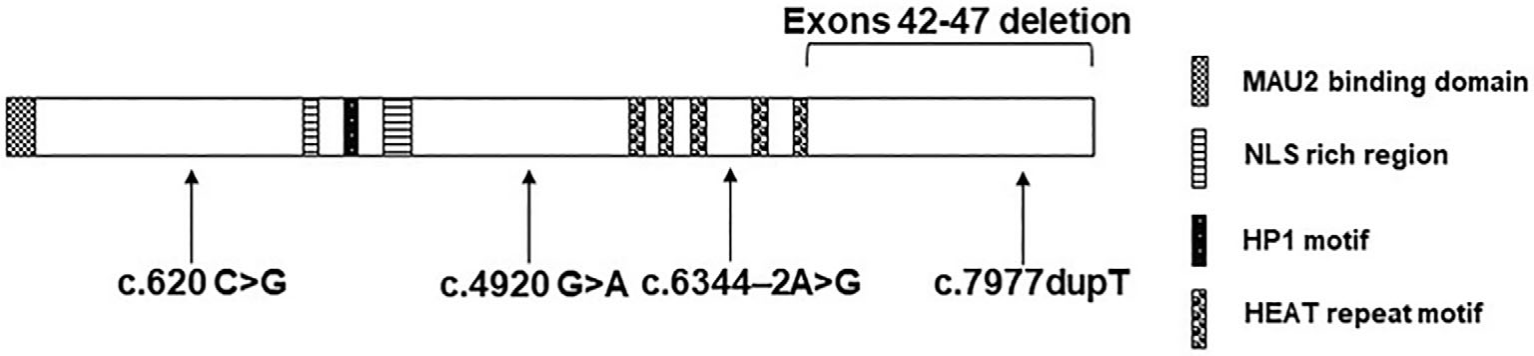
Overview of *NIPBL* variants identified in Cornelia de Lange syndrome patients with concomitant cancer development. *NIPBL* consists of 47 exons and the different domains of *NIPBL* are indicated: MAU2 interaction domain, HP1 interaction domain, nuclear localization signal domain (NLS) and HEAT domain consisting of five repeats. The pathogenic variants, c.620 C>G, c.4920 G>A, c.6344–2A>G, c.7977dupT, and exons 42–47 deletion, are distributed along the entire protein. The protein length is not in scale.

The risk of developing BE and EA increases in presence of gastroesophageal reflux disease (GERD). GERD may be seen in a variety of congenital developmental syndromes, and it is the most frequent and severe gastrointestinal complication in CdLS (Kline et al., 2018). In particular, GERD is almost always present in patients with classic phenotype, that is, carrying *NIPBL* pathogenic variants (Huisman et al., 2017; Luzzani et al., 2003; Nizon et al., 2016). It has been suggested that untreated GERD or chronically unrecognized reflux, with the absence of obvious symptoms, may lead to BE over time (Kline et al., 2007). Though BE is reported in the general population overall with an age at onset over 60 years of age, its incidence in the CdLS cohort is higher than expected and it usually appears at an early age (Bonino & Sharma, 2006; DuVall & Walden, 1996; Kline et al., 2007; Luzzani et al., 2003).

In some syndromes virtually all affected subjects develop a tumor, or associated tumors occur more frequently than in the general population (Postema et al., 2021). The present study indicates that there is no increased risk of cancer in patients with CdLS, although *NIPBL* variants may genetically predispose to early BE development in CdLS. This notion is intriguing since CdLS is caused by pathogenic variants in cohesin structural and regulatory genes, which are also associated with cancer development (Adane et al., 2021; Sarogni et al., 2019). It is still unclear why associated tumors occur with such highly variable frequency in malformation syndromes. Tumorigenesis occurs over the course of many years as a consequence of the accumulation of specific mutations. It is likely that further genetic changes are necessary for a fully malignant transformation, beyond cohesin mutations. The mutational combination of germinal variants of cohesin genes with somatic variants in cancer‐prone genes could be related to specific tumors. In this regard, the identification of mutations and/or the gene expression dysregulation of cancer‐prone genes in cells deriving from CdLS patients with concomitant cancer would support this notion. Some limitations do not allow us to draw a definitive conclusion. In fact, most CdLS patients are young, and reliable data for middle‐aged and older individuals are not currently available. Further studies in larger populations need to be carried out in order to unravel the link between CdLS, cohesin complex, and cancer.

## AUTHOR CONTRIBUTIONS

Maria M. Pallotta and Maddalena Di Nardo reviewed the literature and collected data, Juan Pie, Feliciano J. Ramos, Frank J. Kaiser, Ilaria Parenti, Raoul C. M. Hennekam, and Antonie D. Kline provided data, Antonio Musio took the main lead in writing the manuscript. All authors discussed the results and contributed to the final manuscript.

## FUNDING INFORMATION

This work has been supported by the Associazione Italiana per la Ricerca sul Cancro (AIRC IG23284) to Antonio Musio.

## CONFLICT OF INTEREST

All authors declare that they have no conflicts of interest.

## Data Availability

The data that support the findings of this study are available from the corresponding author upon reasonable request.
